# Mitochondrial Dysfunctions: Genetic and Cellular Implications Revealed by Various Model Organisms

**DOI:** 10.3390/genes15091153

**Published:** 2024-09-01

**Authors:** Monika Stańczyk, Natalia Szubart, Roman Maslanka, Renata Zadrag-Tecza

**Affiliations:** Institute of Biology, College of Natural Sciences, University of Rzeszow, 35-959 Rzeszow, Poland; ms118429@stud.ur.edu.pl (M.S.); ns118430@stud.ur.edu.pl (N.S.); rmaslanka@ur.edu.pl (R.M.)

**Keywords:** mitochondria, mitochondrial dysfunction, model organisms

## Abstract

Mitochondria play a crucial role in maintaining the energy status and redox homeostasis of eukaryotic cells. They are responsible for the metabolic efficiency of cells, providing both ATP and intermediate metabolic products. They also regulate cell survival and death under stress conditions by controlling the cell response or activating the apoptosis process. This functional diversity of mitochondria indicates their great importance for cellular metabolism. Hence, dysfunctions of these structures are increasingly recognized as an element of the etiology of many human diseases and, therefore, an extremely promising therapeutic target. Mitochondrial dysfunctions can be caused by mutations in both nuclear and mitochondrial DNA, as well as by stress factors or replication errors. Progress in knowledge about the biology of mitochondria, as well as the consequences for the efficiency of the entire organism resulting from the dysfunction of these structures, is achieved through the use of model organisms. They are an invaluable tool for analyzing complex cellular processes, leading to a better understanding of diseases caused by mitochondrial dysfunction. In this work, we review the most commonly used model organisms, discussing both their advantages and limitations in modeling fundamental mitochondrial processes or mitochondrial diseases.

## 1. Introduction

**Figure 1:**
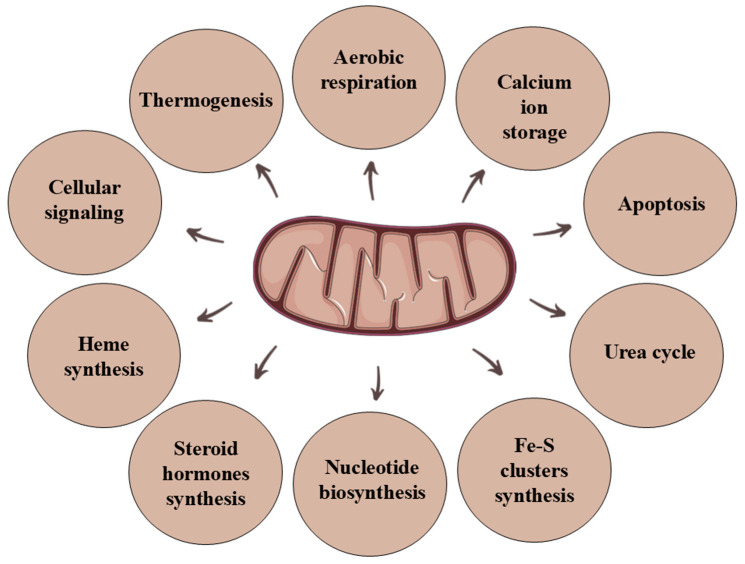
Selected functions of mitochondria.

Mitochondria are well-known intracellular organelles surrounded by two protein-lipid membranes and found in cells of all eukaryotic organisms, including animals, plants, protists, and fungi [1]. However, according to the leading theory of the endosymbiotic origin of mitochondria, they come from prokaryotes. It is assumed that aerobic bacteria that survived endocytosis were incorporated into primitive eukaryotic organisms as endosymbionts [2]. One significant feature of mitochondria is their capacity to interconnect and form an extensive network, thus they should not be viewed as individual organelles. The structure of mitochondria is closely related to their multiple functions. They have a distinct double membrane (outer and inner) surrounding the intermembrane space and the innermost part of mitochondria is called the matrix [3]. The outer mitochondrial membrane is a barrier that isolates the organelles while ensuring contact with the environment. It is relatively easily permeable and contains numerous porins that allow the transport of various substances through the membrane [4]. Unlike the outer membrane, the inner membrane does not contain porins and is much more selective regarding substance permeability. It only allows free diffusion of oxygen and carbon dioxide and water transport [5]. The inner membrane forms mitochondrial cristae, containing electron transport chain complexes and the ATP synthase complex [3]. Although most of the proteins necessary for mitochondria are encoded by the nuclear genome, they contain their own genetic material (mtDNA), replicated independently of nuclear DNA, making them semi-autonomous organelles. The mtDNA encodes select electron transport chain proteins and ATP synthase subunits. In the case of yeast, these are genes for cytochrome c oxidase subunits (*COX1*, *COX2*, and *COX3*), apocytochrome b (*COB*), and three subunits of ATP synthase (*ATP6*, *ATP8*, and *ATP9*) [6,7]. The yeast mtDNA also encodes one ribosomal protein (*VAR1*), large and small rRNAs, and 24 tRNAs [7]. The primary role of mitochondria is their involvement in obtaining cellular energy. They are responsible for generating most cellular ATP through the citric acid cycle and oxidative phosphorylation. However, their functions are not only limited to aerobic respiration; mitochondria are also involved in several other processes, including calcium ion storage, the urea cycle, thermogenesis, heme synthesis, and fatty acids β-oxidation [8] (Figure 1). Ongoing research is being conducted to thoroughly understand the interdependence of mitochondrial functions and the consequences of potential disorders due to numerous cellular functions performed by mitochondria. For this purpose, it is necessary to use model organisms that allow for the analysis of mitochondrial function in a physiological state, as well as the creation of a model of mitochondrial diseases.

## 2. Model Organisms Used in Studies of the Cellular Role of Mitochondria

Model organisms play a key role in unraveling the complexity of mitochondrial biology and understanding the intracellular functions of these organelles. Organisms used in mitochondrial research range from unicellular organisms, such as baker’s yeast *Saccharomyces cerevisiae*, to multicellular organisms, such as *Caenorhabditis elegans*, *Drosophila melanogaster*, *Danio rerio*, and *Mus musculus*. Each of these organisms has particular advantages useful in mitochondrial research, but there are also limitations resulting from their specificity or the complexity of their body structure. These features, as presented in Table 1, may determine the scope of research in which they can be used.

**Figure 2:**
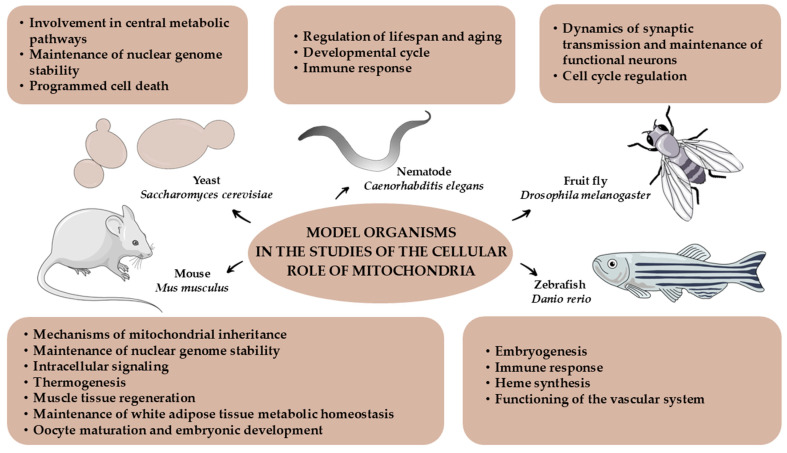
Intracellular roles of mitochondria and the model organisms used to study them.

The use of individual model organisms varies, but they share the common goal of understanding fundamental mitochondrial processes and dissecting conserved pathways that regulate mitochondrial functions (Figure 2).

Research using baker’s yeast *Saccharomyces cerevisiae* mainly focuses on the cellular level due to its unicellular nature. They have contributed to the understanding of the role of mitochondria in cellular amino acid metabolism, including their participation in biosynthetic pathways, mainly by providing metabolic intermediates used as precursors in synthesis [1]. It has been shown that mitochondria are involved in the biosynthesis of many amino acids, especially those synthesized from pyruvate or α-ketoglutarate. They are also involved in the synthesis of fatty acids [22], ceramides [23], and phospholipids that constitute the mitochondrial membranes, i.e., phosphatidylethanolamine and cardiolipin [24]. Research conducted on this model organism also revealed the role of mitochondria in maintaining the stability of the nuclear genome [25]. It was shown that a mitochondrial genetic defect leads to impaired DNA repair processes, which results in a high mutation rate in the nuclear genome of yeast cells [25]. In turn, studies of the mitochondrial proteome have shown that mitochondrial proteins, apart from aerobic respiration, are responsible for the regulation of ion transport [26], the synthesis of ubiquinone [27], the synthesis and assembly of iron-sulfur clusters [28], and protein quality control [29]. Moreover, research in yeast has also contributed to determining the role of mitochondria in programmed cell death [30].

Research conducted with the nematode *Caenorhabditis elegans* contributed to establishing the relationship between mitochondrial function and the regulation of lifespan and the aging process. They also demonstrate that the number of mtDNA copies changes during the development cycle, and the content of the embryo is much smaller than that of the adult. In the later larval stages, there is a rapid increase in the number of mtDNA copies, which is probably related to sexual maturation and reproduction [31]. Moreover, studies involving *C. elegans* allowed us to determine the role of mitochondria in the context of immune responses. Various mitochondrial processes, including mitochondrial surveillance mechanisms, the mitochondrial unfolded protein response (UPR), mitophagy, and ROS production, have been shown to contribute to the immune response and the acquisition of pathogen resistance in *C. elegans* [32].

The fruit fly *Drosophila melanogaster* as a model organism has played a key role in understanding mitochondrial dynamics, primarily due to the discovery, in *Drosophila* sperm, of a protein involved in mitochondrial fusion called mitofusin Fzo1 [33]. This has resulted in an understanding of the function of other proteins related to mitochondrial dynamics and has contributed to defining the role of these organelles in maintaining functional neurons and synaptic transmission. It has been shown that a mutation in the gene encoding Drp1p, a protein involved in the fission of the outer mitochondrial membrane, leads to defects in synaptic transmission [34]. Studies involving *D. melanogaster* have also contributed to the understanding of the role of mitochondria in cell cycle regulation due to their involvement in enforcing the G1/S cell cycle checkpoint under energy deficiency conditions [35].

The participation of *Danio rerio* in mitochondrial research allows us to determine the role of mitochondria in embryogenesis. It has been shown that the use of inhibitors of respiratory complex I or II induced developmental abnormalities, while inhibition of respiratory complex III had a lethal effect in *D. rerio* embryos [36]. This model organism has also been used in studies on mitochondrial dynamics and their relationship with the immune system. The association between mitochondrial metabolic processes and inflammatory responses results from mitochondrial involvement in phagocytosis and efferocytosis. Mitochondria are involved in phagocytosis, mainly through the uncoupling protein Ucp2, and in efferocytosis, due to mitochondrial fission mediated by the Drp1 protein. These proteins induce the release of calcium from the endoplasmic reticulum, which is necessary for the formation of phagosomes and the elimination of apoptotic cells by macrophages [37]. *D. rerio* proved useful in studies on the role of mitochondria in heme synthesis, which have shown that mitoferrin-1 (Mfrn1p), an iron transporter, plays a key role in mitochondrial iron homeostasis. Mfrn1p imports iron from the mitochondrial intermembrane space into the mitochondrial matrix, enabling the biosynthesis of heme groups and iron-sulfur clusters. The mitoferrin-1 defects cause anemia and arrest of erythroid maturation due to insufficient mitochondrial iron uptake [38]. Additionally, research involving *D. rerio* allowed us to understand the role of mitochondria in the vascular system by regulating the properties of endothelial cells. In this case, the action of mitochondria is associated with the adjustment of the intensity of ATP synthesis depending on Ca^2+^ ions and/or ROS signaling in response to external factors [39].

The mouse *Mus musculus*, due to its high physiological and genetic similarity to humans, is considered a particularly useful model for studies of complex physiological processes, such as mitochondrial metabolism, signaling pathways, and the consequences of their dysfunction. The mouse model has also played a key role in dissecting the role of mitochondria in multifactorial diseases. It has been shown, among others, that mitochondria play a key role in maintaining metabolic homeostasis in white adipose tissue cells, due to their involvement in adipogenesis, adipokine secretion, lipogenesis, fatty acid esterification, BCAA (branched-chain amino acid) catabolism, and lipolysis [40]. Moreover, the large number of mitochondria in brown adipose tissue cells is related to their role in thermogenesis. Its mechanism is related to the action of the Ucp1 protein, which uncouples electron transport in the respiratory chain, thereby blocking ATP synthesis and dissipating energy in the form of heat [41]. Studies involving *M. musculus* have also shown that mitochondria are necessary for optimal oocyte maturation and embryo development due to the provision of energy released during aerobic respiration. The mitochondrial energy supply of oocytes is particularly important during early embryonic development because glycolysis is limited during oocyte maturation and early preimplantation embryo development until the blastocyst stage [42]. Other studies have highlighted the role of mitochondrial dynamics in embryonic development. It has been shown that the mitofusins Mfn1 and Mfn2, which coordinate mitochondrial fusion, are necessary for the proper embryonic development of *M. musculus.* Mutations that disrupt their synthesis are lethal [43], and embryonic fibroblasts lacking Mfn1p or Mfn2p display highly fragmented mitochondria. The mouse model makes it possible to determine the influence of mitochondria on maintaining the stability of the nuclear genome [44], as well as the role of mitochondria in the regeneration of muscle tissue. It has been shown that mitochondrial dysfunction affecting the activity of the respiratory chain limits the release of energy needed for muscle regeneration [45]. Additionally, studies conducted on a mouse model revealed the mechanism of mitochondrial inheritance in mammals [46].

## 3. Mitochondrial Dysfunctions

**Figure 3:**
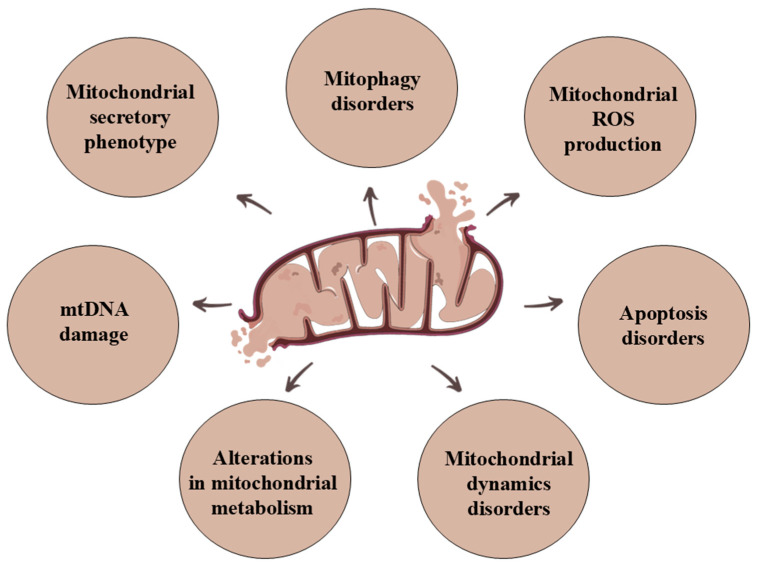
The main symptoms of mitochondrial dysfunction.

Proper functioning of mitochondria is essential to maintain homeostasis in most eukaryotic cells. However, due to the variety of functions of mitochondria, their disorders can cause several abnormalities within the cell and also disrupt the functionality of the entire organism. Mitochondrial dysfunction is defined as the inability to generate the proper amount of ATP and metabolites or transport proteins [47]. This may be due to respiratory chain disorders, mutations in mtDNA, or defective mitochondrial dynamics. Their occurrence may be manifested by many symptoms of mitochondrial dysfunction (Figure 3).

One such symptom is oxidative stress, which may be caused by significantly increased levels of reactive oxygen species (ROS) generated as a by-product of the respiratory chain and oxidative phosphorylation. It is assumed that most ROS is generated by complexes I and III of the respiratory chain [48]. Due to the highly reactive nature of ROS and the main site of their generation, mitochondrial macromolecules (lipids, membrane proteins, oxidative phosphorylation enzymes, and mtDNA) are particularly exposed to the harmful effects of free radicals. Direct damage of mitochondrial proteins reduces their functionality, e.g., by reducing their affinity for substrates or coenzymes [48]. In the case of mitochondrial DNA, ROS can contribute to the formation of various types of mutations, especially since mtDNA exhibits a higher rate of mutagenesis than the nuclear genome due to limited DNA repair mechanisms. Mutations in mtDNA lead to disturbances in the biosynthesis of mitochondrial proteins, disruptions in the electron transport chain, and chemiosmosis, which results in impaired oxidative phosphorylation and energy generation [49]. To counterbalance these changes and prevent ROS generation, cells reduce oxygen consumption, but this action simultaneously further decreases ATP synthesis [50]. Moreover, it has been shown that mtDNA mutations are the basis of mitochondrial diseases in humans, and their presence is characteristic of aging cells and the course of many age-related diseases [51].

Mitochondrial dysfunctions may also be the result of disturbances in the dynamic balance of mitochondria, which leads to a marked change in the morphology and functionality of these organelles [52]. On the one hand, it has been shown that highly differentiated mitochondria are present in cells deficient in fusion proteins, which may contribute to a reduction in their functionality [53]. These cells also exhibit cellular defects such as reduced growth and cellular respiration rates [54]. On the other hand, dysfunctions of the mitochondrial fission process lead to the formation of a heterogeneous population of organelles with a highly variable distribution of mtDNA, varied ability to produce ATP, increased ability to generate ROS, and increased susceptibility of cells to apoptosis [55]. Furthermore, fission dysfunction limits mitophagy, leading to the accumulation of damaged mitochondria inside the cell.

## 4. Consequence of Mitochondrial Dysfunctions

### 4.1. Cellular Senescence and Aging Process

Due to their diversity and participation in complex metabolic pathways, mitochondria are crucial for cellular functioning, and their dysfunction is directly related to the etiology of many diseases. Moreover, mitochondrial dysfunction and the disruption of mitochondrial metabolism are considered to be one of the main causes of the aging process as well as age-related diseases [56,57].

**Figure 4:**
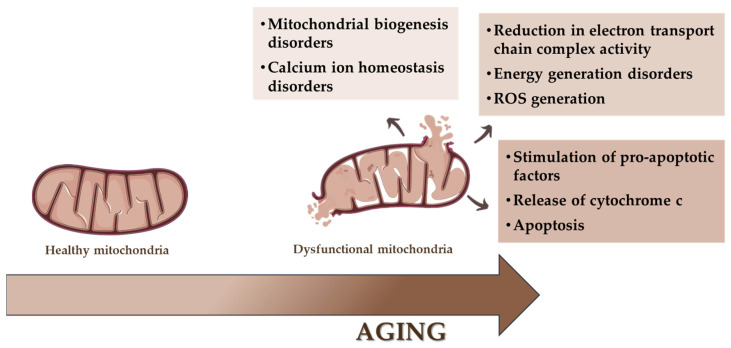
Mitochondria in cellular senescence and aging processes.

It has been observed that, in senescent cells, mitochondria show several significant changes at the structural and functional levels. There can be many reasons for this situation. One of them is reactive oxygen species. Published by Denham Harman in the 1950s, the free radical theory of aging (FRTA) assumed that ROS generated during metabolic processes in mitochondria contribute to the damage of cellular macromolecules, leading to mitochondrial dysfunction and, consequently, to cellular senescence and aging [58,59]. In addition to ROS-derived consequences, mitochondria may contribute to cellular aging through impaired mitophagy, mitochondrial DNA damage, changes in metabolism, or the secretion of specific substances [60], as shown in Figure 4.

Excessive ROS production in the cell can contribute to cellular senescence by inducing the formation of double-stranded DNA breaks. This has been demonstrated, among others, in studies assessing the effect of deletion of the superoxide dismutase 2 (*SOD2*) gene in mice. A deficiency of Sod2p (mitochondria-located enzyme catalyzing the transformation of toxic superoxide into hydrogen peroxide and diatomic oxygen) induced a number of double-stranded DNA breaks in the nuclei of mouse skin cells. In this case, it turned out that a significantly greater number of double-stranded DNA breaks were noted even in very young mice (about 20 days of age) in comparison to mice of the same age but with the functional enzyme. The number of old cells in mice increased due to the lack of the *SOD2* gene and reduced mitochondrial activity [61]. However, mitochondrial ROS may also contribute to telomeres shortening, a characteristic of senescent cells [62]. Studies conducted on human fibroblasts indicate that reducing the amount of ROS produced by mitochondria limits the telomere’s shortening and extends lifespan [63]. The involvement of ROS in cellular senescence was also confirmed in studies carried out using both mice and *C. elegans*. Sod2p deficiency in mice connective tissue cells has been shown to cause accelerated cell senescence and abnormalities in other tissues, e.g., bones, muscles, and skin [64]. In turn, other studies on *C. elegans* have shown that deletion of the *SOD2* gene extends the lifespan of this organism [65].

An increase in the number of mtDNA mutations is also observed with age [66,67]. Most studies on the effects of changes in mtDNA expression have been conducted in mouse models [51]. Mice with damaged mtDNA develop a premature aging syndrome resulting from progressive respiratory chain dysfunction. The changes are caused by the accumulation of mtDNA point mutations as well as the deletion of linear mtDNA molecules. These linear fragments are often removed during mtDNA strand replication [68]. The first signs of premature aging can be observed in mice with damaged mtDNA around 25 weeks of age and include graying of the coat, kyphosis, impaired weight gain, baldness, osteoporosis, and reduced fertility [69]. Experimental studies have also been conducted to investigate whether the mtDNA mutation burden in germlines influences the subsequent rate of aging and the emergence of the aging-related phenotype. Mice with mtDNA mutations inherited from their mothers showed accelerated signs of aging and had a shorter lifespan compared to mice whose mothers did not have mtDNA mutations [70].

In addition, the mitophagy aspect is not without significance for the aging process. Senescent cells are characterized by an increased content of mitochondria and a larger mitochondrial mass, and experimental studies have shown that the level of mitophagy decreases with cell age [71,72]. Studies conducted using a mouse model showed that the level of mitophagy was high in 3-month-old mice and significantly lower in 21-month-old mice [72]. The relationship between mitophagy and longevity has also been confirmed by studies using *C. elegans*. Increased mitophagy has been shown to extend the lifespan of *C. elegans* due to the reduced accumulation of damaged mitochondria in cells [73].

Senescent cells are also characterized by numerous changes in the dynamics and organization of mitochondria, including disturbances in the fusion and fission of these organelles [60]. It is widely accepted that damaged mitochondria can combine with normal ones and thus disrupt their functioning. There may also be disturbances in the process of mitochondria fission, which is necessary to isolate damaged parts of the mitochondrial network and their degradation [74]. Moreover, senescent cells are characterized by numerous changes in mitochondrial metabolism, one of which is an increased ADP to ATP ratio resulting from the decreased efficiency of oxidative phosphorylation [60]. The amount of extra-mitochondrial circulating mtDNA has also been shown to increase with age, which is associated with the increased expression of many pro-inflammatory cytokines and chronic inflammation [71]. The information presented above gives some insight into both how mitochondrial dysfunction can affect the cell’s senescence and the aging process of an organism and also the contributions of the model organisms in understanding the role of mitochondria in these processes. Table 2 summarizes the information about the use of model organisms in studies of the role of mitochondrial dysfunction in the aging process.

### 4.2. Mitochondrial Diseases

Mitochondrial dysfunctions lead to diseases defined as mitochondrial diseases. It is a broad group of diseases caused by mutations in genes found in nuclear or mitochondrial DNA necessary for mitochondrial function. These diseases are generally connected with defects in oxidative phosphorylation and disturbances in ATP production necessary for the proper functioning of cells, especially the nervous or muscular system, due to their high energy demand. For this reason, the symptoms of mitochondrial diseases most often concern functions controlled by the nervous or muscular system, such as muscle weakness or various neurological deficits [84,85]. Due to the involvement of mitochondria in central metabolic pathways and the connection of these organelles with intracellular signaling networks, mitochondrial dysfunctions relate to several neurodegenerative diseases, diabetes, cardiovascular diseases, obesity, and cancer development. The usage of model organisms allows for the study of the biology and function of mitochondria, as well as enabling the modeling of many diseases, contributing to the understanding of their etiology and testing new therapeutic solutions. Selected genetic-derived diseases associated with mitochondrial dysfunction are presented in Table 3, which also notes the model organism used to model the relevant diseases. This is important because, considering both the variety of model organisms and the complications of their structure, not all of them will meet the requirements for the model of a given disease.

## 5. Conclusions

Due to the very important role of mitochondria in maintaining the metabolic and functional efficiency of cells, their dysfunctions impair metabolism and energy generation, leading to many diseases. The development of knowledge on the functioning of mitochondria in physiological and pathological states requires the use of appropriate research techniques, but above all, an appropriate model organism.

The most commonly used model organisms are *C. elegans*, *D. melanogaster*, *Danio rerio*, as well as *Mus musculus*. Their usefulness is confirmed by genome sequencing, which showed that they share 60–70% of gene homology with humans. Nevertheless, it is worth emphasizing that the selection of a model organism should be preceded by an analysis of its opportunities in the scope of modeling the mitochondrial processes or mitochondrial diseases. Each model has its own specific features, which on the one hand can be advantageous e.g., possibility of living without functional mitochondria as in yeast *S. cerevisiae*, but on the other hand, it may be potentially limiting for certain type of studies, e.g., specific physiological adaptation to the environment due to poikilothermy as in the case of *D. rerio*. However, it is worth highlighting that model organisms have made significant contributions to defining the functions and consequences of mitochondrial dysfunction. This is important because understanding the role of mitochondria in cells and explaining the molecular mechanisms underlying their functions and dysfunctions is of great importance not only for deepening knowledge but also for applications, as it allows for the testing of new drugs and therapeutic solutions in the course of mitochondrial diseases or age-related diseases.

## Figures and Tables

**Table 1:** Selected model organisms used in studies of the cellular role of mitochondria.

Organism	Description	Advantages of the Model Organism	Limitations of the Model Organism	References
Yeast *Saccharomyces cerevisiae*	Unicellular fungi belonging to the *Ascomycetes* class Cells with oval shape and diameter 5–10 µm Reproduction by budding Widely used in industry and cell biology as model organisms	Simple cultivation Completely sequenced genome Collections of deletion mutants available Life cycle with haploid and diploid forms enabling the analysis of genetic complementation High susceptibility to genetic manipulation As facultative aerobes, they can maintain viability without functional mitochondria, making them valuable tools for studying mitochondrial-dependent cellular processes The homoplasmic colonies within several divisions	Absence of respiratory complex I Inability to model diseases at the scale of an organ or a complex multicellular organism	[9,10,11,12]
Nematode *Caenorhabditis elegans*	Free-living, non-parasitic nematode Elongated, round body up to 1 mm in length and 80 μm in diameter Complex life cycle Lifespan is 2–3 weeks A constant number of post-mitotic cells organized into five main tissues that make up the digestive tract, epithelium, reproductive system, and nerve and muscle data	Small body size High reproductive rate Easy and low cost of cultivation Possibility of long-term cryopreservation Completely sequenced genome High degree of homology of mitochondrial proteins with mammals Possibility of gene knock-out using RNA interference Self-fertilization in hermaphroditic forms allows obtaining a genetically identical population Transparent body allows fluorescent tags to be tracked at all stages of the life cycle	Less mtDNA copies per cell than in mammals Lacks one of the 37 mtDNA-encoded genes found in humans Lack of DNA methylation The relatively simple anatomical structure and presence of only post-mitotic cells limit the scope of research	[13,14,15,16]
Fruit fly *Drosophila melanogaster*	A small insect from the order of flies (*Diptera*) The development cycle lasts 10 days, from the moment of laying the egg to the formation of an adult Lifespan ranges from 60 to 80 days Widely used in genetic research	Short life cycle High reproductive rate Distinct sexual dimorphism and a wide variety of phenotypes in adults Completely sequenced genome Approximately 75% of human disease-related genes have functional homologs in the *D. melanogaster* genome	Limited possibilities for studying the long-term effects of mutations due to a short lifespan Limited possibilities in transferring results from studies to mammals due to differences in physiology and anatomy	[17,18]
Zebrafish *Danio rerio*	Tropical, freshwater fish from the *Cyprinidae* family Used to model many human diseases related to mitochondrial dysfunction	Small body size Low cultivation cost Ease of genetic manipulation Rapid embryonic development Large number of offspring produced Completely sequenced genome High degree of genetic similarity to humans Transparent embryos enable the study of mitochondrial morphology in vivo	The living environment is different from most mammals and the different physiology of some elements of the respiratory or reproductive system limit the scope of research	[19]
Mouse *Mus musculus*	A small mammal from the order of rodents (*Rodentia*) Relatively short reproductive cycle, pregnancy lasts about 3 weeks, sexual maturity at the age of 6–8 weeks	Small body size, facilitating breeding Relatively short reproductive cycle with numerous litters Genetic and physiological similarities to humans Inbred strains, whose individuals are isogenic, are used to conduct research	When modeling human diseases that involve the induction of oxidative stress, aging, inflammation, or neurodegenerative diseases, its higher ROS generation than in humans should be taken into account	[20,21]

**Table 2:** Model organisms in studies of the role of mitochondrial dysfunction in cellular senescence and aging processes.

Model Organism	Mitochondrial Aspect Related to the Aging Process	References
Yeast *Saccharomyces cerevisiae*	Changes in the morphology of the mitochondrial network Increased mitochondrial ROS generation	[75,76]
Nematode *Caenorhabditis* *elegans*	Antioxidant protection disorders, mitochondrial superoxide dismutase (Sod2p) deficiency The impact of increased mitophagy on lifespan extension Increased accumulation of mitochondria with age Changes in the structure of mitochondria	[65,73,77]
Fruit fly *Drosophila* *melanogaster*	Changes in the general structure of mitochondria, and the structure of mitochondrial crests Mitochondrial ROS formation; overexpression of mitochondrial superoxide dismutase and lifespan extension Changes in oxygen consumption by mitochondria depending on the age of individuals Disorders of mitochondrial dynamics	[78,79,80,81]
Zebrafish *Danio rerio*	The influence of the phospholipid composition of the mitochondrial membrane on the aging process mtDNA quality tests in individuals of different ages Mitochondrial integrity disorders Changes in the number of mtDNA copies in cells of aging individuals Disorders of mitochondrial dynamics (mitochondria fusions and fissions) Reducing the frequency of mitophagy Reduction in the expression of mitochondrial superoxide dismutase (*SOD2*) gene Mitochondrial dysfunction as a cause of aging-related oculopathy, a very common disease worldwide that can lead to visual impairment and blindness	[82,83]
Mouse *Mus musculus*	Antioxidant protection disorders, mitochondrial superoxide dismutase (Sod2p) deficiency, formation of mitochondrial ROS Mutations and damages of mtDNA, both point mutations and deletions of larger fragments Disturbances in the function of the catalytic subunit of mitochondrial DNA polymerase The impact of the germline burden of mtDNA mutations on the subsequent rate of aging and the appearance of the aging-related phenotype, Disorders of the mitophagy process Disorders of mitochondrial dynamics, morphology of the mitochondrial network, impact of deficiency of proteins responsible for mitochondrial fission (PGAM5, DRP1)	[61,68,69,70]

**Table 3:** Selected diseases connected with mitochondrial dysfunctions.

Disease	Genetic Change	Mitochondrial Dysfunctions	Sign and Symptoms of Disease	Model Organism for Studying Disease	References
Diseases caused by mutations in mtDNA					
MELAS syndrome	*MT-TL1* gene mutation	Disturbances in the assembly of proteins into respiratory chain complexes Disorders of oxidative phosphorylation and decrease in ATP synthesis	encephalopathy, lactic acidosis (due to increased glycolysis)	*Saccharomyces cerevisiae*, *Danio rerio*, *Mus musculus*	[86,87,88]
MERRF syndrome	*MT-TK* gene mutation	Decrease in the specific aminoacylation capacity of tRNA-Lys Disorders of mitochondrial protein synthesis Oxidative phosphorylation disorders	progressive myoclonic epilepsy, muscle weakness, and muscle cells appear as frayed fibers	*Danio rerio* *Mus musculus*	[89,90]
Leber’s hereditary optic neuropathy (LHON)	*MT-ND1*, *MT-ND4*, *MT-ND4L*, *MT-ND6* genes mutation	Defects of complex I of the respiratory chain Reduction of mitochondrial membrane potential Decreased ATP synthesis Increased ROS generation	loss of optic nerves due to degeneration of retinal ganglion cells and their axons	*Danio rerio* *Mus musculus*	[86,91,92]
NARP syndrome	*MT-ATP6* gene mutation	Disturbances in the structure and function of ATP synthase Decreased ATP synthesis	neuropathy, ataxia, retinitis pigmentosa	*Saccharomyces cerevisiae*	[93,94]
Chronic progressive external ophthalmoplegia syndrome (CPEO)	deletions in mtDNA	Reduction of mitochondrial membrane potential Decreased ATP synthesis Increased ROS generation cytochrome c oxidase deficiency	myopathy, ptosis, retinitis pigmentosa, central nervous system dysfunction	*Danio rerio*	[91,95]
Diseases caused by mutations in nuclear DNA					
Charcot-Marie-Tooth disease type 2A (CMT2A)	*MFN2* gene mutation	Mitochondrial fusion disorders	degeneration and loss of axons of myelin fibers in peripheral nerves	*Saccharomyces cerevisiae*, *Danio rerio*, *Mus musculus*	[96,97,98]
Alpers-Huttenloch disease	*POLG* gene mutation	Reduction in the functionality of polymerase γ, which is involved in mtDNA replication and repair	progressive loss of cognitive and motor skills	*Caenorhabditis elegans*, *Danio rerio*	[99,100]
Friedreich’s ataxia	*FXN* gene mutation *(encoding frataxin)*	Disorders of frataxin synthesis Iron accumulation in mitochondria Disturbances in the activity of proteins containing iron-sulfur complexes Increased oxidative stress	progressive ataxia of gait and limbs	*Saccharomyces cerevisiae*	[101,102]
Leigh syndrome	mutations of genes encoding proteins of the respiratory chain complexes	Disturbances in the assembly of proteins into respiratory chain complexes Defects in the functioning of complexes I and IV of the respiratory chain Decreased ATP synthesis	necrotic changes in the basal ganglia, brainstem and midbrain, hypotonia, epilepsy, ataxia, lactic acidosis	*Saccharomyces cerevisiae*, *Drosophila melanogaster*, *Danio rerio*, *Mus musculus*	[86,91,103,104]
Barth syndrome	*TAZ* gene mutation *(encoding tafazzin)*	Tafasin deficiency limits the synthesis of cardiolipin, which is a component of the inner mitochondrial membrane	decreased muscle tone, dilated cardiomyopathy, neutropenia	*Saccharomyces cerevisiae*, *Drosophila melanogaster*, *Mus musculus*	[105,106,107]
